# Overall Alteration of Circadian Clock Gene Expression in the Chestnut Cold Response

**DOI:** 10.1371/journal.pone.0003567

**Published:** 2008-10-29

**Authors:** Cristian Ibañez, Alberto Ramos, Paloma Acebo, Angela Contreras, Rosa Casado, Isabel Allona, Cipriano Aragoncillo

**Affiliations:** Centro de Biotecnología y Genómica de Plantas, Departamento de Biotecnología, Universidad Politécnica de Madrid, E. T. S. Ingenieros de Montes, Madrid, Spain; Purdue University, United States of America

## Abstract

Cold acclimation in woody plants may have special features compared to similar processes in herbaceous plants. Recent studies have shown that circadian clock behavior in the chestnut tree (*Castanea sativa*) is disrupted by cold temperatures and that the primary oscillator feedback loop is not functional at 4°C or in winter. In these conditions, *CsTOC1* and *CsLHY* genes are constantly expressed. Here, we show that this alteration also affects *CsPRR5*, *CsPRR7* and *CsPRR9*. These genes are homologous to the corresponding *Arabidopsis PSEUDO-RESPONSE REGULATOR* genes, which are also components of the circadian oscillator feedback network. The practically constant presence of mRNAs of the 5 chestnut genes at low temperature reveals an unknown aspect of clock regulation and suggests a mechanism regulating the transcription of oscillator genes as a whole.

## Introduction

Circadian clocks allow organisms to adapt to periodic environmental changes in light and temperature. In plants, circadian clock performance increases growth, survival and competitive advantage [1], [2]. Although the clock components do not seem to be conserved among kingdoms, clock mechanisms in different organisms are remarkably similar [3]–[5]. In the model plant *Arabidopsis*, it was initially proposed that circadian rhythms are based on a feedback loop in which two partially redundant proteins, LHY (late elongated hypocotyl) and CCA1 (circadian clock associated 1), negatively control their own synthesis by inhibiting the expression of the positively regulating TOC1 (timing of cab, chlorophyll a/b binding protein, expression 1) transcription factor [6]. However, current evidence indicates that the *Arabidopsis* oscillator comprises several interlocking feedback loops, comparable to those identified in the circadian systems of other eukaryotes [7]–[9]. Besides having established roles for the primary clock genes *LHY*, *CCA1* and *TOC1* in the oscillator feedback network, experimental data point to the participation in this mechanism of several *PSEUDO-RESPONSE REGULATOR* (*PRR*) genes, belonging to the same family as *TOC1*/*PRR1* [10]–[14]. This family consists of five members that are expressed after dawn in the sequential order *PRR9→PRR7→PRR5→PRR3→TOC1* [15]. A feedback loop between *PRR9/PRR7* and *LHY*/*CCA1* was initially proposed whereby LHY and CCA1 trigger the expression of *PRR9* and *PRR7*, and the corresponding PRR proteins feed back to regulate *LHY* and *CCA1* expression [11], [13]; *PRR5* was later proposed to also participate in such a loop [12]. Accordingly, computational models of the *Arabidopsis* circadian oscillator include this feedback loop between *PRR9/PRR7* and *LHY*/*CCA1* [16], [17].

In a recent attempt to elucidate the role played by low temperatures in the onset of winter dormancy in woody plants, we showed that circadian clock behavior in the chestnut tree is disrupted in response to cold [18]. The chestnut genes *CsTOC1* and *CsLHY*, which are homologous to essential components of the circadian oscillator in *Arabidopsis*, were observed to cycle daily during vegetative growth as expected. However, during winter, the presence of high non-oscillating levels of *CsTOC1* and *CsLHY* mRNAs indicates alteration of the circadian clock. In addition, we were able to induce a similar disruption by chilling (4°C) chestnut seedlings [18]. To determine the extent to which this chestnut clock disruption affects other elements of the oscillator feedback network, we investigated the behavior of chestnut *PRR* genes during winter dormancy and in response to cold. Here, we report that the interrupted circadian behavior observed previously in *CsTOC1* and *CsLHY* expression is also true of *CsPRR5*, *CsPRR7* and *CsPRR9*.

## Results and Discussion

### The Circadian Behavior of the Chestnut *PRR5*, *PRR7* and *PRR9* Genes

**Figure 1:**
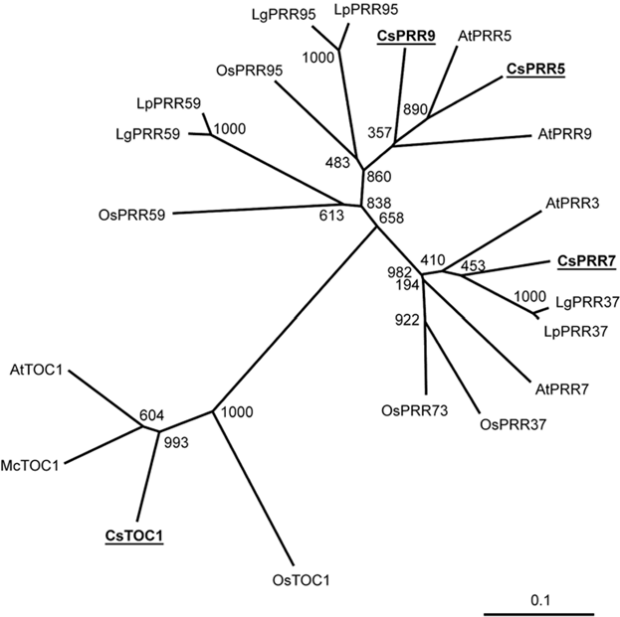
Phylogram of PRR proteins. A non-rooted neighbor-joining phylogenetic tree of PRR proteins was constructed using amino acid sequences of the pseudo-receiver domain (55). Species identifiers are: At, *Arabidopsis thaliana*; Cs, *Castanea sativa*; Lg, *Lemna gibba*; Lp, *Lemna paucicostata*; Mc, *Mesembryanthemum crystallinum*; Os, *Oryza sativa*. The following are the accession numbers for the proteins: AtTOC1 (AF272039); AtPRR3 (BAB13744); AtPRR5 (BAB13743); AtPRR7 (BAB13742); AtPRR9 (BAB13741); CsTOC1 (AY611028); CsPRR5 (ABV53464); CsPRR7 (ABV53463); CsPRR9 (ABV53465); LgPRR37 (BAE72700); LgPRR59 (BAE72701); LgPRR95 (BAE72702); LpPRR37 (BAE72697); LpPRR59 (BAE72698); LpPRR95 (BAE72699); McTOC1 (AAQ73525); OsTOC1 (BAD38854); OsPRR37 (BAD38855); OsPRR59 (AK120059) (KOME database); OsPRR73 (BAD38856); OsPRR95 (BAD38857). Numbers at each branch point indicate the bootstrap replicates (out of 1000) giving rise to the topology.

**Figure 2:**
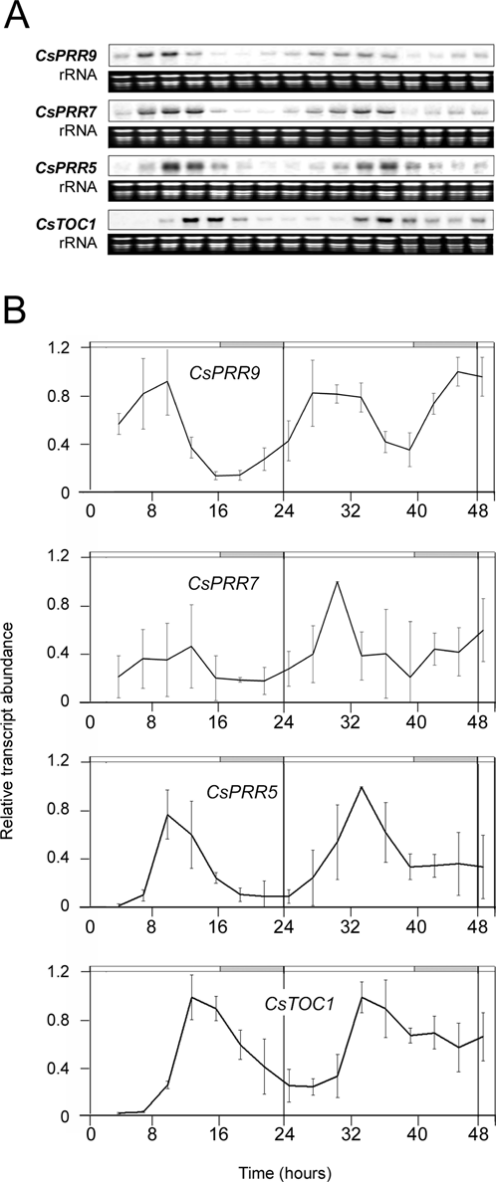
*CsPRR* gene expression levels in chestnut leaves under LL conditions. *CsPRR* gene expression rhythms observed in leaves taken from 16- to 24 week-old chestnut seedlings grown under standard conditions (LD, 22°C) subsequently transferred to conditions of LL and 22°C. Samples were collected at 3-h intervals. (A) Northern blot analysis. The rRNA loading reference was detected by staining gels with ethidium bromide. (B) Quantitative RT-PCR analysis. Relative transcript abundances are shown in the graphs. Data are means from three biological replicates. The open and shaded bars above the graphs represent subjective day and night lengths, respectively.

To help clarify the behavior of the chestnut circadian clock response to cold, additional members of the *PRR* gene family were identified by screening a chestnut stem cDNA library. Three full-length cDNA clones, homologous to *Arabidopsis PRR* genes were isolated. Each clone encoded a polypeptide containing the two domains characteristic of the *Arabidopsis* PRR protein family (PRR1, PRR3, PRR5, PRR7 and PRR9): the pseudo-receiver domain and the CCT (CONSTANS, CONSTANS-LIKE, and TOC1) motif. To explore possible genetic relationships between these chestnut polypeptides and the PRRs previously characterized in *Arabidopsis* and other species [15], [19]–[21], we constructed a phylogenetic tree (Figure 1). The topology of the phylogram revealed three groups of PRR proteins, as has been observed by Murakami *et al.* [19]. One of these groups, which includes CsTOC1/CsPRR1, comprises the PRR1 proteins of the different species. The other two groups each included representatives of two PRR proteins: PRR5 and PRR9 in one group, and PRR3 and PRR7 in the other. In these last two groups, the PRR proteins of rice, *Lemna gibba* and *Lemna paucicostata* were designated two numbers (37 or 73, and 59 or 95), since it was difficult to estimate which PRR from rice or the two *Lemna* species corresponded to which *Arabidopsis* PRR [19], [21]. Of the three chestnut pseudo-response regulators identified in the present study, two were found to group with the proteins PRR5 and PRR9. These PRRs were denoted CsPRR5 and CsPRR9 based on the relative distances shown in the phylogram to *Arabidopsis* PRR5 and PRR9. The third chestnut PRR we could infer from the clones isolated, grouped within the PRR3 and PRR7 protein class. Given the characteristics of the phylogram and the fact that we did not isolate the gene coding for the other PRR protein of this group in the chestnut, it is difficult to ascribe the corresponding homology to this CsPRR. We opted for tentatively designating it as CsPRR7 rather than CsPRR3 based on the similar circadian expression pattern of the gene to that shown by the *Arabidopsis PRR7* gene (Figure 2; see below) [15].

To test the circadian behavior of the *CsPRR* genes, we analyzed their expression in leaves collected from 16- to 24 week-old chestnut seedlings grown under continuous light (LL) conditions in a temperature-controlled (22°C) growth chamber over a 48 h period. RNA samples were analyzed by Northern blot hybridization using probes specific for each gene *CsTOC1/PRR1*, *CsPRR5*, *CsPRR7*, and *CsPRR9*. These probes were sequences of the non-conserved region of *PRR* family genes, flanked by the pseudo-receiver and CCT domains. We observed the diurnal oscillation of transcript levels of the *CsPRRs* over an approximate 24 h period (Figure 2). These chestnut PRR transcripts started to accumulate after subjective dawn in the order *PRR9* and *PRR7→PRR5→TOC1*. Despite the concurrent appearance of *PRR9* and *PRR7* mRNAs, *PRR9* mRNA peaked earlier than *PRR7* mRNA. This expression order of the *CsPRR* genes resembles that of the *Arabidopsis PRR* genes (*PRR9→PRR7→PRR5→PRR3→TOC1*) more than the order noted in the monocotyledons rice or *Lemna* spp. [15], [19], [21]. For example, circadian analysis of *OsPRR* in rice also indicates their sequential expression but in a different order: *OsPRR73* and *OsPRR37→OsPRR95* and *OsPRR59→OsPRR1* [19]. Differences among species in the temporal order of *PRR* gene expression could be essential, given the direct role these genes play in the machinery of the circadian oscillator [11]–[14]. In particular, the feedback loop proposed among *PRR9*, *PRR7*, and possibly *PRR5* and *LHY*/*CCA1*, which is thought to form part of a multiple-loop network [7]–[9], could be affected by these differences. The expression interval of each *PRR* could also be a determining factor for their participation in clock output pathways. The findings of a recent study suggest that PRR9, PRR7 and PRR5 regulate flowering time in *Arabidopsis* through the CONSTANS- dependent pathway [22]. Interestingly, a genetic complementation analysis in which the rice genes *OsPRR1* and *OsPRR37* were introduced into the corresponding *Arabidopsis* loss-of-function mutants (*toc1* and *prr7* respectively) revealed that these genes are only partially interchangeable between these species [23].

### The Circadian Behavior of *CsPRR* Genes is Disrupted in Response to Low Temperatures

**Figure 3:**
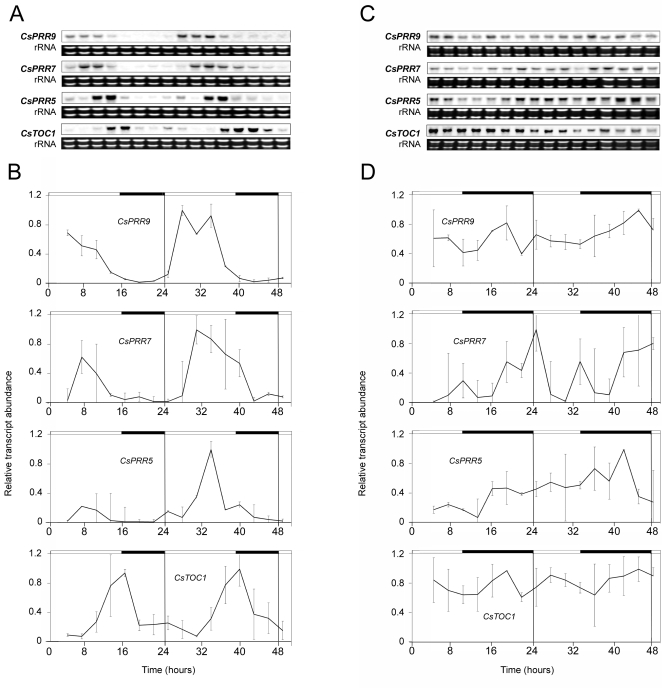
*CsPRR* gene expression in stems of adult chestnuts under different seasonal conditions. Stems (second-year branch internodes) collected in June (A and B) and December (C and D). Samples were collected at 3-h intervals. (A and C) *CsPRR* northern blot analysis. The rRNA loading reference was detected by staining gels with ethidium bromide. (B and D) Quantitative RT-PCR analysis. Relative transcript abundances are shown in the graphs. Data are means from two biological replicates. Open and filled bars above each graph represent natural day and night lengths, respectively, as provided by the National Institute of Meteorology, Madrid.

When we addressed whether the circadian behavior of *CsPRR5*, *CsPRR7* and *CsPRR9* was modified during winter dormancy or in response to cold, similar changes were observed to those described previously for the central oscillator genes, *CsLHY* and *CsTOC1* [18]. We examined the expression of the *CsPRR* genes in adult chestnuts grown under natural light and temperature conditions in Zarzalejo, Madrid (4°11′W, 40°35′N): first in June, which is when vegetative growth takes place, and then in December, when temperatures are low and these trees are in a state of endodormancy. In samples collected in June, oscillatory expression patterns were observed for the four *CsPRR* genes. The characteristics of their fluctuations were consistent with those described above for plantlets exposed to continuous light. This behavior was noted both in stem and leaf samples (Figures 3A, 3B, and S1). In contrast, in stem samples collected in December, the circadian expression of the *CsPRR* genes was modified. Thus, rather than exhibiting typical daily cycles, *CsPRR* mRNA levels remained consistently high (Figure 3C and 3D). Hence, the previously described altered expression of *CsTOC1* and *CsLHY* in winter was similarly shown by *CsPRR5*, *CsPRR7* and *CsPRR9*.

**Figure 4:**
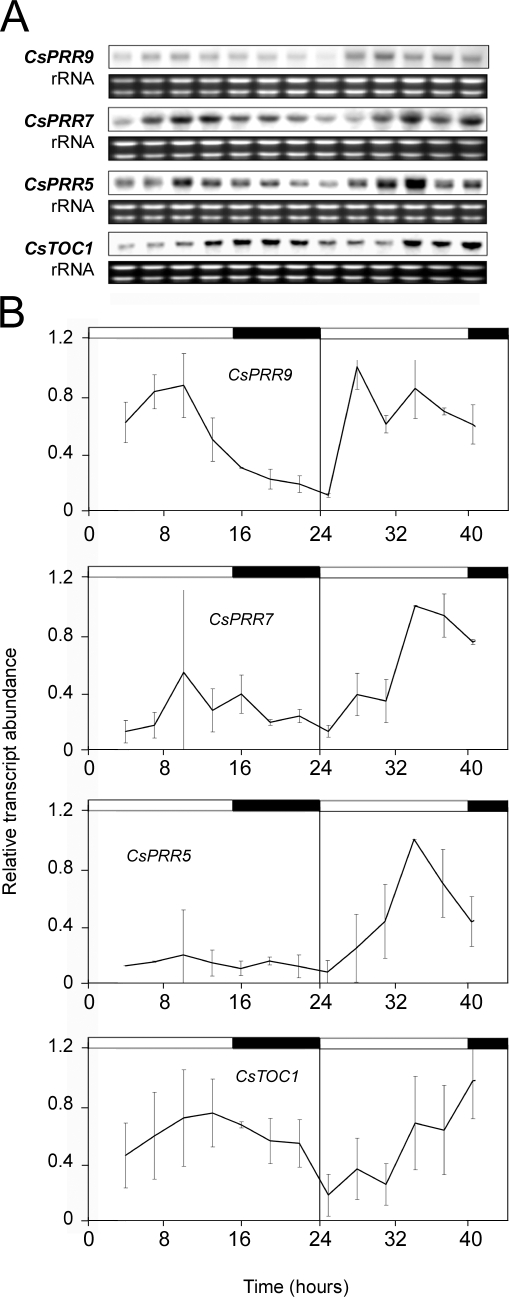
Recovery of circadian *CsPRR* gene expression in endodormant chestnut seedlings. *CsPRR* gene expression rhythms observed in stems from 11 month-old endodormant chestnut plants grown under natural conditions in Madrid and subsequently transferred to controlled-environment chambers under conditions of 22°C and LD for 1 week. Samples were collected at 3-h intervals. (A) Northern blot analysis. The rRNA loading reference was detected by staining gels with ethidium bromide. (B) Quantitative RT-PCR analysis. Relative transcript abundances are shown in the graphs. Data are means from two biological replicates. Open and filled bars above each graph indicate lights on and lights off, respectively.

We were also able to confirm that, as for *CsTOC1* and *CsLHY*, this alteration is not intrinsic to the state of endodormancy. Endodormancy is caused by plant endogenous factors and, once established, no growth can be achieved until a chilling requirement has been satisfied. In order for bud break to occur, plants need to be exposed to low temperatures for a cumulative number of hours. Thus, endodormant plants that had not yet satisfied the chilling requirement, were transferred to growth chambers kept at 22°C and subjected to a long day (LD) photoperiod (16 h light / 8 h dark). In these conditions, the plants remain in a state of endodormancy yet they gradually lose their characteristics exclusive to the cold response. After one week at 22°C, expression levels of the *CsPRR* genes in stem tissue had recovered their circadian rhythm (Figure 4).

**Figure 5:**
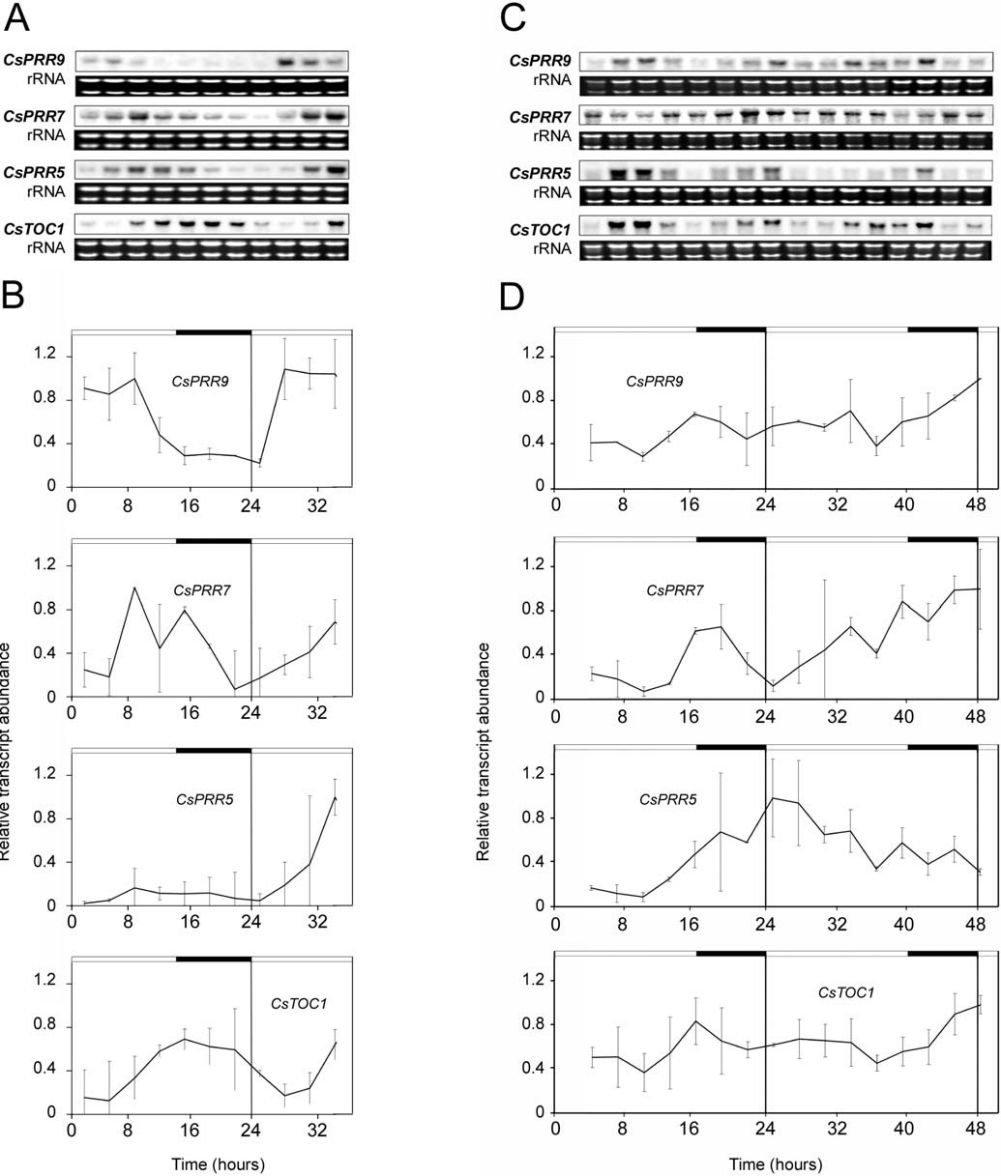
*CsPRR* gene expression in the stems of chestnut seedlings grown under different temperature conditions. The figure shows *CsPRR* blot analysis and mRNA abundances. (A and B) Stems from seedlings grown under conditions of LD and 22°C. (C and D) Stems from seedlings grown under standard conditions (LD, 22°C) and subsequently subjected to one week of LD at 4°C. Samples were collected at 3-h intervals. (A and C) *CsPRR* northern blot analysis. The rRNA loading reference was detected by staining gels with ethidium bromide. (B and D) Quantitative RT-PCR analysis. Relative transcript abundances are shown in the graphs. Data are means from two biological replicates. Open and filled bars above each graph indicate lights on and lights off, respectively.

To establish whether exposure to low temperatures was sufficient to interrupt the circadian behavior of the *CsPRR* genes, experiments were performed on 16- to 24 week-old chestnut seedlings. *CsPRR* expression patterns in stems and leaves collected from seedlings grown under the conditions 22°C and LD were compared to expression patterns in seedlings subjected to 4°C and LD conditions for 1 week. At 22°C, the mRNAs of the 4 *CsPRR* genes indicated their cyclic expression with specific circadian oscillation both in stem and leaf samples. In contrast, in the plants exposed to low temperatures, this oscillatory behavior was interrupted (Figures 5 and S2). These effects of cold were characterized by the presence and not by the absence of the *CsPRR* mRNAs over time, as occurs in adult trees during winter.

These results indicate that low temperatures greatly modify the expression of at least 5 of the genes potentially involved in the oscillator mechanism of the chestnut circadian clock. This cold response in the chestnut may translate to all woody species. Indeed, expression levels of clock oscillator genes in *Populus alba* have also been observed to not show circadian cycling in winter (our unpublished results). In contrast, the *Arabidopsis* clock seems to exhibit different behavior in response to cold. Cycling of the mRNA levels of genes under circadian control (*CAB* and *CCR2*, cold-circadian rhythm-RNA binding 2) has been observed in *Arabidopsis* seedlings grown for 5 days at 4°C under a light/dark photoperiod [24] and, recently, Bieniawska *et al.* [25] have shown that under continuous light conditions, cold disrupts the circadian expression of *Arabidopsis* oscillator genes, while in normal diurnal light-dark conditions cold only reduces the amplitude of cycles of clock components. The different responses to low temperatures shown by the circadian clocks of *Arabidopsis* and the chestnut could be attributable to differences in the clock mechanism or in the regulation of a shared mechanism. It is difficult to predict which mechanisms of control could be affected by cold, and besides the possibility of an influence on transcriptional level control other alternatives should be considered. Studies on clock gene regulation mechanisms in plants at the posttranscriptional and posttranslational levels are currently underway [26]–[30]. In effect, of the four *PRR*s examined in this work, three (*TOC1*, *PRR5* and *PRR7*) are now known to be regulated at the protein level in *Arabidopsis* [14], [26], [29]. Moreover, the circadian expression of *TOC1* has been recently correlated with clock-controlled histone acetylation rhythms of its promotor [31].

Cold disruption of the chestnut circadian clock in normal diurnal light-dark conditions indicates that the cold acclimation process in woody plants may have special features compared to similar processes in herbaceous plants. Differences between species are hardly surprising when one considers that trees have had to adapt to two types of thermal stress: the fluctuating temperatures endured during the growing season and the continuous temperature drop that occurs in winter. Stopping of the clock is likely to have an impact on the general physiology of the plant, since this molecular oscillator is known to regulate major metabolic pathways, and over 10% of genes in *Arabidopsis* are under circadian control [32]–[34]. Moreover, the circadian clock participates in cold signaling pathways in *Arabidopsis* by gating the low-temperature induction of CBFs (CRT/DRE, C-repeat/drought-responsive, binding factor) and modulating low-temperature Ca^2+^ signals [35], [36]. Welling and Palva [37] have shown that in addition to their role in cold acclimation during the growing season, CBFs are involved in the regulation of cold tolerance during overwintering in birch.

It has long been established that the photoperiod controls the induction of winter dormancy processes in most trees growing in temperate climates [38], [39]. Recently Böhlenius *et al.* have shown that the CO/FT (constans/flowering time locus T) regulatory module, which controls flowering time in response to variations in daylength in annual plants, also regulates short-day-induced growth cessation [40]. However, in order to attain an advanced state of dormancy, low temperatures are also required and it is known that changes such as leaf senescence and abscission are not provoked by a short day photoperiod alone, but also require exposure to cold [41], [42]. In fact, in the apple tree and other species of the family Rosaceae, cold induces dormancy regardless of photoperiodic conditions [43]. Although winter clock disruption does not seem to maintain dormancy, since in endodormant plants transferred to conditions of 22°C and a LD, standard *PRR* gene expression cycling resumes, the initial changes induced by the first low autumn temperatures could trigger the onset of endodormancy. In addition, stopping of the circadian clock linked to low winter temperatures could in part explain the extensive remodeling of meristem transcriptome observed in the vascular cambium of poplar during the transition from growth to dormancy [44]–[47].

A similar circadian clock response to the cold has been observed in the ruin lizard (*Podarcis sicula*), a hibernating ectothermal vertebrate. At low temperatures (6°C), the cycling expression of two clock genes (*Per2* and *Clock*) in this animal is diminished in peripheral clocks with a characteristic increase in basal expression levels [48], [49]. The basic mechanisms of clock function in plants and animals are similar, although their oscillator genes are unrelated. This parallelism between two such evolutionary distinct organisms suggests that the stopping of the circadian clock in response to cold could be part of a general adaptive strategy that enables living organisms that undergo dormancy or hibernation to survive the winter. Recently, the interruption of the molecular circadian clock in the European hamster during hibernation has also been described; the clock genes *Per1*, *Per2*, and *Bmal1* being constantly expressed in the suprachiasmatic nucleus during deep torpor [50]. Interestingly, clock disruption in the three organisms results in increased expression of oscillator genes, suggesting a positive role of these components during low temperature periods.

In summary, our findings indicate that in the chestnut, low temperatures disrupt the canonical cyclic expression of the circadian oscillator genes *CsPRR5*, *CsPRR7*, and *CsPRR9*, as previously observed by us for *CsTOC1/PRR1* and *CsLHY*. Concurrent changes in the expression of these five genes forming part of several oscillator feedback loops, point to control mechanisms not yet elucidated by current *Arabidopsis* circadian clock models.

## Materials and Methods

### Plant Material and Growth Conditions

In field experiments, stem samples (2-year-old branch internodes) and leaves were harvested from adult European chestnut trees (*Castanea sativa* Mill.) in Zarzalejo, Madrid (4°11′W, 40°35′N). Samples were collected in the months of June (22.8°C average temperature; 15 h, 5 min average day length) and December (4.9°C average temperature; 9 h, 16 min average day length). Controlled-environment experiments were performed using 16–24 week-old chestnut seedlings in growth chambers under the conditions described in Ramos *et al.* [18]. For the long day (LD) trials, seedlings were grown at 22°C and subjected to a 16 h light/8 h dark (16∶8) photoperiod. Exposure to cold (4°C) was undertaken for a week under the same light regime. Continuous light (LL) experiments were performed on plants that had been grown under conditions of LD and 22°C and thereafter subjected to LL from dawn. Endodormancy experiments were performed as in [18]. Chestnut plants grown in natural conditions enter a state of endodormancy at the end of November. Before satisfying the chilling requirement, plants were transferred to a growth chamber at 22°C under a 16 h light/8 h dark photoperiod and kept for 1 week in these conditions before sample collection. After subjecting the plants to the different treatments, specimens were collected at 3-h intervals. Each experiment was performed at least twice.

### Isolation of cDNA Clones

Chestnut *CsPRR5*, *CsPRR7* and *CsPRR9* cDNA clones were isolated from a λUni-ZAP XR cDNA library following standard procedures [51]. The library was constructed using chestnut stem poly (A)^+^ RNA isolated from plants in winter [18]. To detect *CsPRR* clones other than *TOC1*, a full length *CsTOC1/PRR1* clone was used as probe on two replicate membranes, one under high and the other under low stringency hybridization and washing conditions. After discarding the common spots as *TOC1* clones, three different *CsPRR* clones were detected corresponding to fragments of the genes *CsPRR9*, *CsPRR7* and *CsPRR5*. To obtain the *CsPRR9* full-length cDNA clone, the corresponding fragment was used as probe under high-stringency hybridization and washing conditions. Probes were labeled with [*α*-^32^P] dATP using a random-primed DNA labeling kit (Roche Applied Science, Indianapolis). Full-length cDNA clones for *CsPRR7* and *CsPRR5* were obtained using the “BD SMART RACE cDNA Amplification” kit (Clontech, Mountain View, CA) according to the manufacturer's instructions.

### Northern Blot Expression Analysis

Total RNA was obtained from chestnut stems and leaves as described in [52], separated on 1.2% agarose gels with 2.2 M formaldehyde [53], and subsequently transferred to Hybond-XL nylon membranes (GE Healthcare Bio-Sciences Corp., Piscataway, NJ). ^32^P-labeled DNA probes used to detect each specific mRNA were designed to only span the non conserved region of each pseudo response regulator gene. The probes were PCR amplified using appropriate sets of forward and reverse primers:

*CsPRR5* forward 5′-GGCAAATCGTTTCCAAGTGA-3′ and
*CsPRR5* reverse 5′-TAGAAGAGTTGACAAGGACATA-3′,
*CsPRR7* forward 5′-GAAGACATCGGGATGTGCAA-3′ and
*CsPRR7* reverse 5′- CCTGAACACAGCTAGTGCC-3′,
*CsPRR9* forward 5′-GCTTCCTCGCATTGCTACAG-3′ and
*CsPRR9* reverse 5′-AACAACAAAGCCAGGCATCG-3′.

A gene fragment spanning *CsTOC1* nucleotides 1380–1591 that specifically recognizes the *TOC1*/*PRR1* member of the pseudoresponse regulator (*PRR*) gene family was used as in Ramos *et al.* [18]. Northern blot hybridizations were conducted according to the recommendations of the membrane manufacturer. Membranes were washed at high stringency, exposed on storage phosphor screens and visualized in a TYPHOON 9400 phosphorimage scanner (GE Healthcare, Bio-Sciences Corp.). The rRNA loading reference was estimated by staining gels with ethidium bromide.

### Real time RT-PCR Expression Analysis

Total RNA was obtained from chestnut stems and leaves as described in [52] with a modification introduced after LiCl precipitation in which the RNeasy Plus Mini Kit columns from Qiagen were used. This kit includes one step to eliminate any contaminating genomic DNA in the total RNA sample. To check the lack of degradation, RNA was separated by electrophoresis on a formamide-formaldehyde denaturing agarose gel. RNA purity and quantity were checked with a Nanodrop Spectrophotometer. For each sample, single stranded cDNA was synthesized from one microgram of total RNA using the Superscript III First-Strand Synthesis SuperMix for qRT-PCR (Invitrogen). This mix includes oligo (dT)_20_ and random hexamers. First-strand cDNA was synthesized in a 2720 thermocycler (Applied Biosystems). Gene-specific primers were designed using Primer Express 2.0 (Applied Biosystems) for the non conserved region of each pseudo response regulator gene as follows:

*CsPRR5* forward 5′-GCAAAACAAAGAAGAATACTTG-3′ and
*CsPRR5* reverse 5′-CTTCACTCCCATGCGTAAG-3′,
*CsPRR7* forward 5′-ATTTGTTAAGTGCGTCCCTTG-3′ and
*CsPRR7* reverse 5′- TTTCCATATTTGTTCCTGAAGC-3′,
*CsPRR9* forward 5′-GAGGTTGTGCCCTTCGGAG-3′ and
*CsPRR9* reverse 5′-ACAAGCATTTTCCTTCAATCTC-3′.
*CsTOC1* forward 5′-ACTTGATGCTTCTGGCTTACCT-3′ and
*CsTOC1* reverse 5′-ATTGTGCTGCTGATGGC-3′
*Cs18S* forward 5′-TCAACTTTCGATGGTAGGATAGTG-3′ and
*Cs18S* reverse 5′-CCGTGTCAGGATTGGGTAATTT-3′

Real time polymerase chain reactions were performed in an optical 384-well plate with an ABI PRISM 7900HT Sequence Detector System (Applied Biosystems), using SYBR Green to monitor dscDNA synthesis [54]. The reaction mixtures contained 1× Power SYBR Green Master Mix reagent (Applied Biosystems), 250 nM gene-specific primers (except *Cs18S*, for which 25 nM gene specific-primers were used) and 0.4 µl of the previously synthesized cDNA (except *Cs18S* for which 0.4 µl of a 25-fold fresh dilution of cDNA was used) in a final volume of 10 µl. The following standard conditions were used in all PCRs: 40 cycles of 95°C for 30 s and 60°C for 1 min. A dissociation step was performed after amplification to confirm the presence of a single amplicon. To estimate variation in the technique, three technical replicates were carried out for each biological replicate. Data were analyzed using SDS 2.2.2 software (Applied Biosystems). To generate a baseline-subtracted plot of the logarithmic increase in fluorescence signal (Δ*R*_n_) versus cycle number, baseline data and the *R*_n_ threshold were detected automatically to obtain Ct (threshold cycle) values. Amplification efficiency for each gene was calculated based on four dilutions of template ranging from 500 ng to 0.5 ng and the equation *E* = 10^−1/slope^-1, with slopes in the range slope = −3.3±0.1 and E = 2. mRNA abundances for each candidate gene were calculated as: relative transcript abundance = *2* ^(−ΔΔCt)^. For the sample chosen as calibrator, ΔΔCt = 0 and therefore the fold-change = 1. Quantified data are shown in the graphs as relative amounts of mRNA. The sample with maximum expression (lower ΔCt) was used as calibrator and 18S ribosomal RNA was used as the reference gene to normalize data. The absence of genomic DNA contamination was checked using Non-Retrotranscriptase controls (RT-) and the absence of environmental contamination using Non-Template Controls (NTC).

## Supporting Information

Figure S1 *CsPRR* gene expression in the leaves of adult chestnuts obtained in June. Leaves were collected in June at 3-h intervals. (A) *CsPRR* northern blot analysis. The rRNA loading reference was detected by staining gels with ethidium bromide. (B) Quantitative RT-PCR analysis. Relative transcript abundances are shown in the graphs. Data are means from two biological replicates. Open and filled bars above each graph represent natural day and night lengths, respectively, as provided by the National Institute of Meteorology, Madrid.
(0.33 MB TIF)

Figure S2 *CsPRR* gene expression in the leaves of chestnut seedlings grown under different temperature conditions. (A and B) leaves from seedlings grown under conditions of LD and 22°C. (C and D) leaves from seedlings grown under standard conditions (LD, 22°C) and subsequently subjected to one week of LD at 4°C. Samples were collected at 3-h intervals. (A and C) *CsPRR* northern blot analysis. The rRNA loading reference was detected by staining gels with ethidium bromide. (B and D) Quantitative RT-PCR analysis. Relative transcript abundances are shown in the graphs. Data are means from two biological replicates. Open and filled bars above each graph indicate lights on and lights off, respectively.
(0.62 MB TIF)
