# Supplementary figures and images for: Overall Alteration of Circadian Clock Gene Expression in the Chestnut Cold Response
Source: PLoS One. 2008 Oct 29;3(10):e3567. doi: 10.1371/journal.pone.0003567 (PMC2569414; doi:10.1371/journal.pone.0003567)

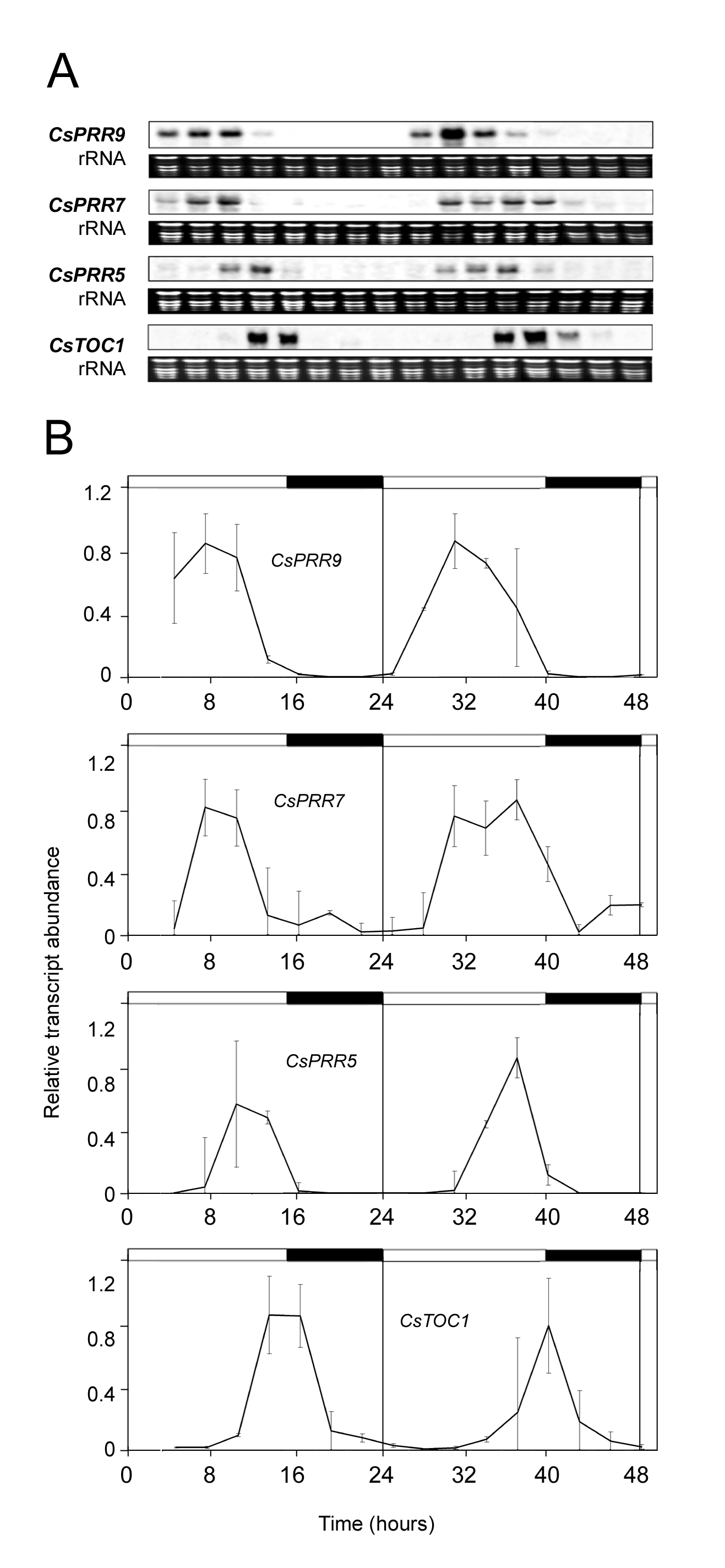

Supplement: Figure S1 — CsPRR gene expression in the leaves of adult chestnuts obtained in June. Leaves were collected in June at 3-h intervals. (A) CsPRR northern blot analysis. The rRNA loading reference was detected by staining gels with ethidium bromide. (B) Quantitative RT-PCR analysis. Relative transcript abundances are shown in the graphs. Data are means from two biological replicates. Open and filled bars above each graph represent natural day and night lengths, respectively, as provided by the National Institute of Meteorology, Madrid. (0.33 MB TIF) [file pone.0003567.s001.tif]

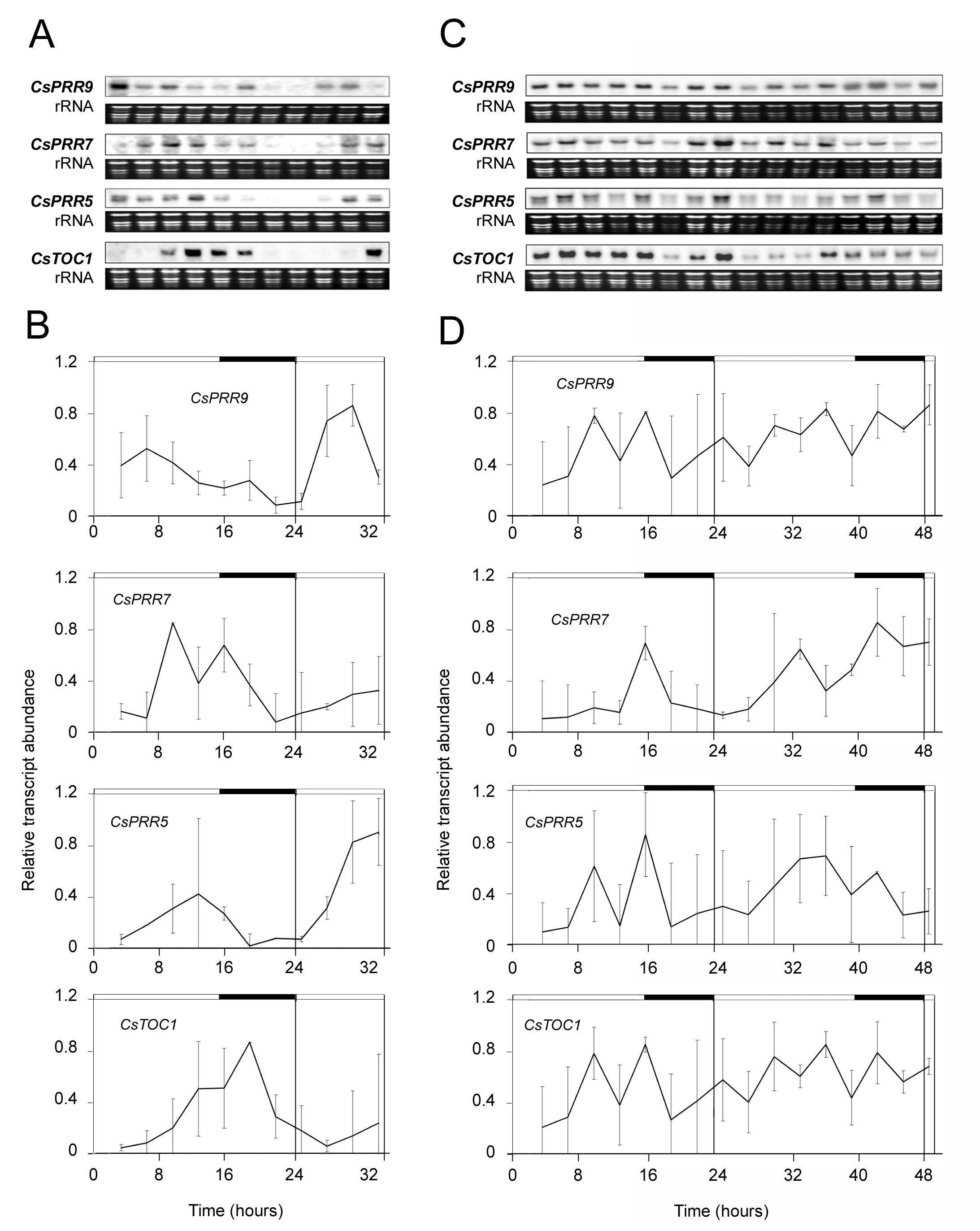

Supplement: Figure S2 — CsPRR gene expression in the leaves of chestnut seedlings grown under different temperature conditions. (A and B) leaves from seedlings grown under conditions of LD and 22°C. (C and D) leaves from seedlings grown under standard conditions (LD, 22°C) and subsequently subjected to one week of LD at 4°C. Samples were collected at 3-h intervals. (A and C) CsPRR northern blot analysis. The rRNA loading reference was detected by staining gels with ethidium bromide. (B and D) Quantitative RT-PCR analysis. Relative transcript abundances are shown in the graphs. Data are means from two biological replicates. Open and filled bars above each graph indicate lights on and lights off, respectively. (0.62 MB TIF) [file pone.0003567.s002.tif]
